# English as a Foreign Language Teachers’ Work Engagement, Burnout, and Their Professional Identity

**DOI:** 10.3389/fpsyg.2022.916079

**Published:** 2022-06-09

**Authors:** Zhaopeng Xing

**Affiliations:** The School of Economics, Xiamen University, Xiamen, China

**Keywords:** burnout, professional identity, work engagement, EFL teachers, education

## Abstract

Teacher-related variables have been considered as determining factors in shaping educational system. Teacher professional identity is also very important construct that affect teachers’ teaching practice Moreover, the positive and negative emotions are considered sporadically in Chinese English as a Foreign Language (EFL) educational contexts; however, and there is a paucity of research in investigating the relationship between work engagement as a positive emotion and teacher burnout as a negative workrelated factor. This review also tried to examine how teachers’ professional identity is affected by teachers’ positive and negative emotions in Chinese contexts. Earlier studies have verified that teacher burnout is significantly correlated with professional identity. The earlier investigations showed the mediating role of job resources, such as job satisfaction and obligation, in the correlation between teacher burnout and professional identity. Moreover, studies have shown a significant negative correlation between teacher burnout and work engagement. Some variables, including personal features, instructive environment, work difficulties, job capital, character strengths, self-efficacy, close relationship with school colleagues, and emotional intelligence, mediate the correlation between teacher burnout and work engagement. Finally, this review specified that teachers’ professional identity is regarded as a critical variable in fostering work engagement. Emotional intelligence was also considered a mediating variable that affected the relationship between teacher professional identity and work engagement. Moreover, the study has pedagogical implications and suggestions for different teacher educators, administrators, and advisors. The ideas can improve their awareness of teacher burnout, professional identity, and work engagement in instructive contexts.

## Introduction

The process of language teaching is a complex one wherein different factors are more or less influential in the quality of instruction. The teacher is considered one of the important factors as an active decision-maker (Freeman, 2002). There is evidence in the literature that teachers’ performance is best predicted by a collection of knowledge, skill, and affective characteristics (Amirian and Behshad, 2016). This means that language teaching is not just limited to technical knowledge and skills. In addition to English language competence, some affective factors also influence the process of language teaching. It should be underlined that instructors have their individual personality traits, principles, reasoning, and reflection that considerably influence their teaching activities in educational contexts (Kim et al., 2019). Among different teacher variables, teacher professional identity, teacher work engagement, teacher burnout, etc., can be mentioned.

One of the affective factors which is effective in language teaching is teachers’ professional identity or the way that teachers define their professional roles. This is considered one of the significant issues in education, which is related to teachers’ commitment (Rezaei, 2018). As argued by Rezaei (2018), many studies have shown the effectiveness of teachers’ professional identity on their performance and professional development. It has even been reported in some studies that learning to teach involves professional identity construction rather than knowledge acquisition (Nguyen, 2008). The concept of teachers’ work engagement, as another variable, in educational contexts, is significant but disregarded in the conventionalized EFL classroom contexts (Zhao et al., 2021). According to Schaufeli et al. (2002), teachers’ work engagement refers to “a positive, fulfilling, work-related state of mind that is characterized by vigor, dedication, and absorption” (p. 202). They argued that dedicated and absorbed instructors can provide inspiring educational contexts in which learners tend to engage in the learning process. Teachers’ work engagement can predict their teaching effectiveness, activities, problem-solving, and job satisfaction (Minghui et al., 2018).

Another affective factor mentioned as influential in language teaching is teacher burnout. Burnout is a feeling experienced in different occupations especially teaching, due to long-term occupational anxiety. However, there are various reasons that anxiety has been referred to as one of the main causes of burnout (Jennett et al., 2003). Burnout is experienced in the form of emotional and mental exhaustion and job stress (Maslach, 1976). Burnout consists of three factors, emotional exhaustion, depersonalization, and reduced personal accomplishment. Emotional exhaustion refers to being emotionally overextended (Brouwers and Tomic, 2000), depersonalization is about becoming cynical and distant from one’s work and from others as a form of coping (Lee and Ashforth, 1990; Taris et al., 2005), and reduced personal accomplishment refers to having a negative evaluation of oneself (Brouwers and Tomic, 2000). The reason why teachers experience burnout very much is that burnout is usually associated with people-oriented occupations (Maslach, 1976). In the same vein, as stated by Seferoğlu et al. (2014), teaching is a profession where teachers come into contact with a lot of burnout, which can be connected with pressure on teachers, training students who deal with many spiritual, behavioral problems, teachers‘ duties, etc. However, despite the importance of teacher-related factors in English teaching, the researcher noticed that this characteristic of English training has not been suitably accentuated. More particularly, while teacher burnout is regarded as a severe problem in educational systems (Blandford, 2000) which may negatively affect teachers’ professional and personal life and deteriorate their teaching efficiency (Carson et al., 2011), to the best knowledge of the researcher, so far, few (if any) studies have dealt with these negative factors as related to some other affective factors including teacher engagement, and teacher professional identity. This review is an attempt to study the investigations that bridge this gap.

## Literature Review

### Teacher Professional Identity

Hashemi et al. (2021) defined identity as interdisciplinary, which is “the style of one’s individuality which coincides with the sameness and continuity of one’s meaning for others in the immediate community” (p. 2). The notion of identity is applied in numerous ways in teaching contexts (Derakhshan et al., 2020). In instructional contexts, Pennington (2015) defined identity as “a construct, mental image or model of what ‘being a teacher’ means that guides teachers’ practices as they aim to enact ‘being a teacher’ through specific ‘acts of teacher identity” (p. 17). According to Barkhuizen (2016), “language teacher identities are multiple, and they change, short-term and over time discursively in social interaction with teacher educators, learners, other teachers, administrators, and the broader community, and in material interaction with spaces, places, and objects in classrooms and institutions” (p. 659). Professional identity is a dynamic construct that impacts teachers’ behaviors in the classroom, their teaching effectiveness, and their sense of wellbeing. It also affects their professional development and helps them to cope with educational changes, bring innovation to the classroom and be creative in their teaching practice (Abednia, 2012), Beijaard et al. (2000) identified three aspects of professional identity: teachers as subject matter experts, teachers as didactical experts, and teachers as pedagogical experts. The results showed that teachers identify themselves mostly as didactical experts, then as pedagogical experts, and least as subject matter experts. Teachers interpret the curriculum and textbooks, choose the style of their teaching, and gain an understanding of students’ learning based on their professional identities. Since teachers’ professional identity experiences are the core of their teaching practice, exploration of teachers’ professional identity has great implications for curriculum reform, classroom teaching, and student learning (Caihong, 2011). Hamachek (1999) states that teachers teach students what they know consciously.

Some variables have been recognized as the most important determinants in forming of teacher professional identity. Marcia (2002) stated that teachers’ capabilities and aptitudes, along with their professional interests in having relationships with learners, are the most prominent issues in the development of educators’ professional identity. He maintained that a profound and reciprocated relationship with the contexts and society, and emotional satisfaction in instruction can form the conceptualization of a teachers’ professional identity. Other demographical variables can also influence teachers’ professional identity. Bukor (2015) stated that teachers’ demographical issues can determine their development of professional identity. Mora et al. (2016) found out that teachers with higher educational levels have a strong professional identity. Andreasen et al. (2019) also found other factors, including educational context, teachers’ beliefs about the role, and school principals’ and administrators’ cooperation, that affect teachers’ professional identity. Beijaard et al. (2000) also believed that teachers who consider themselves as subject matter experts feel that the teaching experience, as a demographical factor, can affect their performance.

Thomas and Beauchamp (2011) stated that teachers’ emotions have mutual relationships with the development of professional identity. Few studies have been done on the effect of teachers’ negative emotions and positive emotions on professional identity (Cheng, 2021). This review examines the studies on the relationship between teacher professional identity, work engagement (as a positive emotion), and burnout (as a negative emotion).

### Teacher Work Engagement

According to Fredricks and Simpkins (2013), engagement is defined as the degree of learners’ and educators’ commitment and investment in their performance. He also maintained that engagement is regarded as an umbrella term that consists learners’ and educators’ degree of devotion, concentration, and inclination to use abilities, approaches, or activities to develop their performance. Louis and Smith (1992) pointed out that “in primary or secondary education, teacher engagement refers to a teacher’s psychological investment in an effort toward teaching the knowledge, skills, and crafts he or she wishes students to master” (p. 120). Raina and Khatri (2015) stated that some factors, such as educational experience, learners’ aptitude, class size, school location, class the school, classroom contexts, classroom management, task management, novelties in educational contexts and instruction, feedback received by learners and principal, interaction with colleagues, and opportunities for cooperation with others are critical in teacher engagement.

Teacher engagement has been investigated in many studies which considered its relationship with their demographical variables. Topchyan and Woehler (2021) found that full-time female educators with higher levels of social involvement with learners have more degrees of work engagement and job satisfaction. They also found a significant correlation between work experience and work engagement.

Benesch (2018) stated that language educators’ feelings can be considered the causes of work engagement. Likewise, Ghanizadeh and Moafian (2010) showed that positive affectivity, including enjoyment and hope, are critical in shaping educators’ engagement. Consequently, the prominence of controlling positive and negative feelings is remarkable in educational contexts where feelings play an important role in adjusting the quality of instruction and engagement. Jennings and Greenberg (2009) mentioned that instructors with high social and affective capabilities could positively discover applied solutions in challenging contexts and build up their engagement. Diener et al. (2020) mentioned that positive feelings affect teachers’ performance in language teaching together with long-term work involvement, positive attitudes, resourcefulness, operative teaching strategies, and teacher-learner rapport. They argued that positive feelings activate upward spirals, since the positive results predict upcoming rises in positive feelings, and result in work engagement and wellbeing. Greenier et al. (2021) showed that teacher wellbeing and emotional regulation strategies significantly correlate with teacher engagement. They argued emotional regulation strategies used by teachers are effective for their involvement in doing educational tasks. Zeng et al. (2019), in their study in the Chinese context, demonstrated that teachers’ growth mindset, wellbeing, and resilience strongly predict job engagement. They also found out that wellbeing and grit mediate the correlation between work engagement and growth mindset. Sonnentag et al. (2008) also found a negative and significant correlation between work engagement and emotional exhaustion. Han et al. (2020) also listed the main reasons for teachers’ less work engagement and exhaustion: teaching difficulties, teaching-research conflict, and new challenges in teacher-learner relationship. This review aims to investigate burnout, as another negative emotion, and its relationship with teacher engagement.

### Teacher Burnout

Maslach (1976) defined burnout as “physical, emotional, and attitudinal exhaustion which leads to a negative attitude toward clients and a decline in the quality of work” (p. 2). Recently, teacher burnout has been widely investigated in the instructional field (Nayernia and Babayan, 2019). This is possibly predictable, as a result of numerous strains and stressors that educators experience (McCarthy et al., 2016). In this situation, the burnout indications will be noticeable in frequent individual and interactive results. For instance, teacher burnout has been related to diminished work capacity, non-attendance, and eventually learners’ weak academic performance (Chang, 2009).

Akbari and Eghtesadi Roudi (2020) listed some causes for teacher burnout, including anxiety, inability to manage classroom management and deal with learners’ troublemaking behaviors, overwork, discontent with the workplace and working position, unfairness, conflict in the workplace, lack of social support, deficiency in feedback and admiration, less involvement in decision making, and less teacher self-sufficiency. Schwab and Iwanicki (1982) also stated that background, individual personality, organizational factors, class size, work environment, and workload are described as the source of teacher burnout. Moreover, the effects of several demographic factors on teacher burnout have been examined. Kara (2020) found a significant correlation between individualized variables such as gender, years of experience, marital status, and burnout. Jamaludin and You (2019) also showed that experience and educational level among males and females demonstrated significant relations with emotional exhaustion, depersonalization, and reduced personal accomplishment. Their findings implicate that school system administrators regard such demographic factors as crucial in recruitment decision-making and retention programs.

Studies have also shown that teacher burnout results in more inadequate academic performance among learners. For example, Madigan and Kim (2021) have demonstrated that teacher burnout is significantly correlated with poorer educational success and less motivation. Some studies have also been done on the relationship between teacher burnout, as a negative emotion, and teachers’ positive emotions, such as self-efficacy, wellbeing, and resilience. Sak (2018) revealed that male teachers have higher levels of organizational suspicion and burnout when measured by the aspects of emotional exhaustion, depersonalization, and individual achievements. On the other hand, Ghasemzadeh et al. (2019) found that teacher burnout is strongly predicted by self-efficacy in Iranian educational contexts. They argued that when educators feel fewer insights about their capability in classroom management, the probability of occupational stress may increase, which builds up emotional exhaustion and depersonalization. Capone et al. (2019) found the significance of burnout in the psychological wellbeing of educators since it mediates the correlation between job variables and teacher depression. Polat and İskender (2018), in their study, have displayed the predictability of teacher burnout by resilience. They argued that teachers with higher levels of resilience, who can appropriately deal with changes, will be exposed to lower levels of burnout.

### The Relationships Among Teacher Professional Identity and Work Engagement, and Teacher Burnout

Few investigations have been done on the relationship between teacher professional identity and work engagement. Van Der Want et al. (2019) found a significant relationship between teachers’ professional identity and work engagement. Butakor et al. (2021) found that teachers’ professional identity is considered an essential issue in instructors’ engagement with their job, which regulates learners’ educational success. They also argued that emotional intelligence positively influenced teacher professional identity and job satisfaction which affected work engagement. Zhao et al. (2019) examined the impacts of instructors’ self-efficacy and work engagement on their professional identity development in multi-cultural regions. They argued that instructors’ viewpoints about instruction and their practical involvement in instruction, were significantly correlated with professional identity. Correa Gorospe et al. (2018) examined the effects that an altering world and risky job conditions can have on newly qualified teachers’ sense of engagement with the children, the school, and the profession in general as part of their professional identity. They argued that having teachers analyze their sense of engagement with the children, their colleagues and the schools, in general, is a strategy for articulating their personal and professional development, for facilitating their capacity to adapt to the endless changes in the teaching profession and for helping them to reformulate their identity as teachers. Ghamoushi and Mohamadi Zenouzagh (2020) asserted that collaborative discussion among EFL teachers can foster their work engagement and professional identity.

Few studies have been done on the relationship between professional identity and burnout among EFL teachers. Fisherman (2015) investigated kindergarten, elementary, and high school educators’ professional identity, and its effect on their burnout. He found out that teacher professional identity ad job experience significantly predict burnout in educational contexts. He justified the results by arguing that there is a difference between role conflict and role ambiguity. He argued that there are three causes of role ambiguity: “(a) absence of clear information regarding areas of responsibility, obligation, and rights; (b) absence of clear information about the alignment of the expectations; (c) absence of available information generated by changes within the organization” (p. 20). He maintained that role ambiguity acts as a predictor of teacher burnout. He asserted that role ambiguity in terms of expectations among teachers is the main reason for the difference in the burnout levels among kindergarten, elementary, and high school educators. Kindergarten teachers have a comparatively well-defined set of expectations. They feel accountable to their parents and supervisors. On the other hand, high school teachers feel more ambiguous about their responsibilities toward learners, parents, public, school principals, etc. the policymakers set ambiguous instructions toward schools and teachers. He believed that “teachers sometimes find themselves in the midst of a conflict between various Ministry of Education functionaries, each pulling in a different direction” (p. 21). Lu et al. (2019) found the mediating role of job satisfaction in the relationship between teacher burnout and teacher professional identity. Their findings also revealed that professional identity and job satisfaction are strong predictors of teacher burnout. They used the job demand-resource model in order to justify their results. They argued that job resources, including work engagement, work satisfaction, and obligation, lead to great results and diminish undesirable consequences. Therefore, high levels of job satisfaction build up the significant and positive impacts of teachers’ professional identity on burnout. Their study implicated that main intervention techniques can be employed to reduce Chinese educators’ burnout. Troesch and Bauer (2017) also found out that teachers’ professional identity is negatively correlated with burnout. They argued that teacher self-efficacy mediates the correlation between teacher burnout and teacher professional identity. Zhao et al. (2020), in another study, found that Chinese teachers’ professional identity, particularly valence and self-presentation, are significantly and negatively correlated with teacher burnout. They argued that once instructors have the feeling that instruction is fascinating, and see the value in education, they are less likely to get bored in their job. They also found out that instructors’ educational background, as a type of teacher variable, can also mediate the correlation between teacher professional identity and burnout. They argued that teachers with higher educational backgrounds do not experience burnout in their work. The deeper educational experience, probably, allows them to have greater academic knowledge and wider perspectives over instruction, and they are eager to operate their knowledge.

Some investigations have been done on the relationship between teacher burnout and teacher engagement. Using Demerouti et al.’s (2001) Job Demands-Resources Model, Hakanen et al. (2006) investigated the effect of burnout and work engagement on their health problems and organizational commitment. They found out that learner misconduct, amount of work, and classroom context induce inappropriate health situations *via* their influence on burnout. Their results indicated that educators’ job regulation, support, information, community, and innovation are correlated with organizational commitment through work engagement. Faskhodi and Siyyari (2018) found a significant and negative correlation between work engagement and burnout. Their study also revealed that vigor, as an element of job engagement, significantly correlated with teacher burnout. They stated that novice educators demonstrated higher levels of burnout. They argued that educators typically start a challenging job with higher levels of enjoyment. However, after a short time of instruction, they cope with the diversity of unpredicted complications for which they are unable to discover support. Moreover, experienced educators are able to employ coping strategies. Salmela-Aro et al. (2019) investigated educators’ job involvement and burnout through employing an individual-based method. Their study indicated that engaged educators had high levels of job and personal resources, including regulation and flexibility, while educators with high levels of burnout and engagement experienced workload. Mojsa-Kaja et al. (2015) compared two groups of teachers, including those who have high levels of burnout and those who are engaged in classrooms. They found that educators with high levels of burnout remarked an incongruity between themselves and the educational contexts. However, engaged educators, compared to educators with high levels of burnout, show their orientation toward educational contexts, and they have less negative emotions and great levels of self-directedness.

In another study, Hultell and Gustavsson (2011) examined the effect of personal features and the instructive environment on teacher burnout and job engagement during the first years of entering educational contexts. They found out that work difficulties, job capital significantly predicted burnout, and work engagement. Their study also revealed that work difficulties had a significant relationship with burnout, while job capitals were significantly associated with teachers’ job involvement. They stated that “it would be methodologically unsound to claim that it would be possible to control for levels of burnout and work engagement during education. The difference in context between education and employment and that burnout and work engagement are psychological states and thus are context-dependent. However, this does not necessarily mean that future burnout and work engagement might not be affected by feelings of stress and psychological strain during education” (p. 94).

Using Maslach’s (1976) Job Burnout questionnaire and Schaufeli et al. (2006) engagement questionnaires, Heidarilaghab and Talepasand (2021) found that teachers’ involvement in educational contexts is a critical issue in reducing burnout. Their study also revealed that character strengths mediate the correlation between job involvement and teacher burnout. Their study indicated that policymakers should regard the development of empowerment programs to enhance job involvement and diminish teacher burnout. Using social cognitive theory, Llorens-Gumbau and Salanova-Soria (2014), in a study, indicated that the obstacles (negative attitudes of learners), facilitators (availability of pertinent materials over instruction), and self-efficacy significantly affected teacher burnout and engagement. Their findings revealed that obstacles are significantly correlated with burnout, which, in turn, is positively associated with self-efficacy. They argued that teachers’ self-efficacy increases available materials which significantly influence work engagement. Their study also confirmed the efficacy of the spiral model. Moreover, their study demonstrated that self-efficacy and facilitators are mutually affected by each other in a longitudinal study. Fiorilli et al. (2019) found out that a close relationship with school colleagues and support mediate the relationship between teacher burnout and work engagement. D’Amico et al. (2020) asserted that teachers’ emotional intelligence mediates the correlation between teachers’ work engagement and burnout. They argued that “educators, who assume that they have competency in evaluating feelings and employing them in positive and adaptive ways, feel more involved at work, more satisfied, and to experience fewer burnout symptoms” (p. 21). Juliana et al. (2021) also conducted a study that proved the mediating role of teachers’ work engagement in the relationship between teacher burnout and Job Demands-Resources Model. Their study revealed that job demands were in a significant negative relationship with work engagement whereas job resources were in a significant positive relationship with work engagement. Furthermore, it is determined that job demands and job resources possessed a significant indirect relationship with burnout, respectively, through work engagement as a mediator. They argued that the negative features of job demands used to make the job difficult for employees to feel engaged, which in turn fades away the feeling of enthusiasm and dedication toward their job.

## Conclusion

This review investigated the relationship between teacher burnout, teacher professional identity, and teacher work engagement. This review showed that teachers with higher levels of professional development are more inclined to have higher levels of work engagement. On the other hand, the enhancement of professional identity can decrease teacher burnout. This review also demonstrated that work engagement can reduce teacher burnout.

## Implications and Suggestions for Future Research

This review includes some pedagogical implications for teacher educators, policymakers, and advisors. In the first place, one of the pedagogical implications of this review is that if language educators tend to increase their work engagement, they should attempt to decrease their emotions of burnout. Another one is that teachers’ challenges at cultivating their professional identity can result in a decrease in their emotions of burnout. Moreover, professional identity plays an important role in the enhancement of teaching efficiency, as it reveals the convergent demands of individuals and society (Deng et al., 2018). In order to build up professional identity, teachers should assess their performance and knowledge about instruction. Teachers also can participate in conferences, do research, and communicate with their colleagues in order to develop their professional identity. During studies, reflection should be encouraged by offering students assignments for the development of professional and personal growth (Ivanova and Skara-Mincāne, 2016). Teachers should face up to their profession, recognize the nature of their profession, have the courage to shoulder the responsibility of education and set up correct professional values. In the process of teaching, they should learn to establish a friendly relationship with students. While guiding and helping students to learn scientific and cultural knowledge, their own professional ability will also be improved.

Teachers should also reduce their burnout in order to increase their efficiency. In order to deal with the sources of burnout and stress, educators can keep on learning new knowledge to foster their required abilities and approaches in the educational contexts, continue cooperating with their associates, and pay attention to their physical wellbeing throughout their occupation. Educators ought to be motivated to observe other institutes to learn extra educational strategies which could improve educational organization and decrease the extent of challenges. Educators should have the knowledge about the reduction of anxiety to prevent the activators of teacher burnout. They can get this knowledge by taking part in conferences, sharing of experiences among educators, or receiving some emotional support from counselors. Long-lasting pressure and anxiety can inexorably exhaust educators, leaving them incompetent in doing their occupation; nevertheless, with the appropriate treatments, we can ultimately eradicate teacher burnout (McCarthy et al., 2016).

Teachers should also increase their work engagement by increasing their motivation and autonomy. Higher levels of teacher self-sufficiency allow teachers to possess a robust feeling of belonging, to meet their needs of affinity. The robust insight of affinity specifies that the educators are in a friendly relationship with both associates and learners. This positive rapport and educational context will enhance educators’ inclination to do additional activities beyond their occupation like assisting to other educators (Runhaar et al., 2013). Teachers with higher satisfaction of relatedness needs will also receive more support from each other, and they are more likely to collaborate with each other, all of which will enhance the overall effectiveness of the school. Consequently, teachers’ view over affinity will develop their job involvement.

To improve teacher professional identity, work engagement, and to reduce teacher burnout, teacher educators and mentors should encourage motivating teachers to involve in their job, so that they can contribute to the soundness and constructiveness of the educational system. Teacher educators can also emphasize instructors to attach importance to the constant academic development and critical thinking to enhance their engagement and professional identity. Instructors should be directed to be well-informed about instructive issues and take advantage of improved learning chances. It is also suggested that teacher educators highlight interaction tools, like mobile applications, which encourage teachers and learners to interact and scaffold that increase work engagement and reduce burnout. They should develop confidence and competence among in-service teachers to entice learners’ interests and engage them in the learning process. Educational policymakers can decrease teacher burnout by holding academic workshops that offer teachers some authentic strategies. They can ask teachers to do their best within varied educational contexts. Policymakers should also provide critical thinking, creativeness, and motivation into the education in classrooms, which encourages work engagement. School principals should provide the opportunity to instructors to determine the individual-based attitude in the course of teaching learners. Besides, school principals ought to vigorously design and establish a diversity of cooperative activities to foster educators’ feeling of belonging to allow educators to experience comfort, enjoyment, companionship, and cordiality of their colleagues in the school. Schools and institutes should pay more attention to instructors’ involvement at work, and it is critical to enthusiastically prepare educators to contribute to the building of school culture and have educators feel they are the controllers of their educational contexts. However, the employment of this approach can boost instruction quality. Once the educational contexts possess high respect and status in the public, the position of educators and their feeling of belonging to the occupation will be concurrently developed.

The importance of professional identity and work engagement can motivate advisors to expand their horizons to identify teachers’ sources of work engagement and identity development. Future studies should aim to replicate results in larger contexts. In future work, investigating teachers’ work engagement, professional identity, and burnout in technology-supported contexts, numerous cultural backgrounds, and among teachers with different educational experiences can be important for future studies. Some investigations need to be done on teachers’ professional identity and work engagement on learner motivation in traditional and virtual contexts. Furthermore, the relationship between teacher proficiency level of foreign language, and its effect on their work engagement, burnout, and professional identity should be considered for the future. Furthermore, case and phenomenological investigations, which provide us the reasons behind teachers’ burnout, development of professional identity, and teacher burnout are required to be done. Some studies should be done on the relationship between positive psychological constructs such as enjoyment, grit, positive affectivity, resilience, and teacher professional development. In addition, future research should examine the roles of negative factors such as anger, and frustration in teacher burnout and work engagement. Some investigations should also be done on the effect of cross-cultural perspectives on teacher burnout and professional identity development. The role of some other factors, such as socioeconomic background, employment standards, and methodology in the development of teacher professional identity ought to be considered in the future. Finally, investigating the relationship among the other teacher characteristics including organizational treatment, self-concept, and work engagement can make for a good research topic.

## Author Contributions

ZX took responsibility for research design and writing, contributed to the article, and approved the submitted version.

## Conflict of Interest

The author declares that the research was conducted in the absence of any commercial or financial relationships that could be construed as a potential conflict of interest.

## Publisher’s Note

All claims expressed in this article are solely those of the authors and do not necessarily represent those of their affiliated organizations, or those of the publisher, the editors and the reviewers. Any product that may be evaluated in this article, or claim that may be made by its manufacturer, is not guaranteed or endorsed by the publisher.
